# Anti-NMDAR encephalitis after resection of melanocytic nevi: report of two cases

**DOI:** 10.1186/s12883-015-0424-z

**Published:** 2015-09-14

**Authors:** Xun-zhe Yang, Li-ying Cui, Hai-tao Ren, Tao Qu, Hong-zhi Guan

**Affiliations:** Department of Neurology, Peking Union Medical College Hospital, Center of Neuroscience, Chinese Academy of Medical Sciences, Beijing, 100730 China; Department of Dermatology, Peking Union Medical College Hospital, Chinese Academy of Medical Sciences, Beijing, 100730 China

**Keywords:** Anti-NMDAR encephalitis, Antibody, Melanocytic nevi

## Abstract

**Background:**

Encephalitis with antibodies against N-methyl D-aspartate receptor (NMDAR) is recognized as a group of antibody-mediated neuropsychiatric syndromes, which occurs with and without a tumor association. Neoplasm may contribute to the pathogenesis of Anti-NMDAR encephalitis in tumor-positive patients. However, the underlying causes in tumor-negative patients are largely unknown. This is the first report, of which we are aware, of two cases of anti-NMDAR encephalitis after the resection of melanocytic nevus.

**Case presentation:**

We describe 2 female patients in their 20s confirmed with anti-NMDAR encephalitis. They shared two points in common: About several weeks (2 weeks and 5 weeks respectively) before the initial symptom, both of them underwent a resection of melanocytic nevi; the screening tests for an ovarian teratoma and other tumors were all negative. A 25 year-old woman presented with seizure, psychiatric symptoms and behavioral change for 2 weeks. Electroencephalogram indicated electrographic seizures. Anti-NMDAR antibodies were all positive in the cerebrospinal fluid and serum. Her symptoms relieved gradually after the treatment with steroids and mycophenolate mofetil. Another patient admitted to our hospital with psychosis, behavioral change and complex partial seizure over a period of 5 months. Electroencephalogram demonstrated generalized slow activities. High titres of anti-NMDAR antibodies were both detected in the cerebrospinal fluid and serum. She responded well to the first-line immunotherapy and got substantial recovery.

**Conclusion:**

Our cases provided an observational link between anti-NMDAR encephalitis and resection of nevi. We postulate that the exposure of certain antigen on nevus cell caused by nevi excision, which might be NMDA receptor or other mimic cross-reactive antigens, may trigger an autoimmune response resulting in encephalitis. This suggested a potential site of antigen exposure triggering the immune response in non-tumor associated anti-NMDAR encephalitis, which may lend support to elucidating the underlying immunopathological mechanisms. Further studies are expected for investigating the expression of NMDA receptor on nevus cell and evaluating the validity of this hypothesis.

## Background

Encephalitis with antibodies against N-methyl D-aspartate receptor (NMDAR) is recognized as a group of antibody-mediated neuropsychiatric syndromes, which occurs with and without a tumor association. Neoplasm may contribute to the pathogenesis of anti-NMDAR encephalitis in tumor-positive patients. Other evidence showed that non-specific systemic infections or vaccinations could act as an adjuvant of the autoimmune response. However, the underlying causes in tumor-negative patients are still largely unknown. This is the first report, of which we are aware, of two cases of anti-NMDAR encephalitis after the resection of melanocytic nevus.

## Case presentation

### Case 1

A 25 year-old woman presented with seizure, psychiatric symptoms and behavioral change for 2 weeks. About 5 weeks before her first symptom, she underwent a resection of nevi on her nose in a local hospital, of which the pathological diagnosis is compound nevus confirmed by the dermatologist. Her family history was unremarkable. The brain magnetic resonance imaging (MRI) showed normal while the electroencephalogram (EEG) indicated electrographic seizures. For her lumbar puncture, the cerebrospinal fluid (CSF) white blood cell (WBC) count was 2/μL; the CSF protein concentration, 54 mg/dl. The CSF-specific oligoclonal bands were weak positive. The serum and CSF samples were tested for the antibodies to cell-surface antigens including NMDAR, leucine-rich glioma inactivated 1 (LGI1), contactin-associated protein 2 (CASPR2), γ-amino-butyric acid-B receptor (GABABR) and alpha-amino-3-hydroxy-5-methyl-4-iso-xazolepropionic acid receptors (AMPAR), using a commercial assay (Catalogue No. FA 112d-1003-1, EUROIMMUN AG, Lübeck, Germany). Anti-NMDAR antibodies were all positive in the CSF and serum. Screening with an ultrasonographic examination of her ovaries and a computed tomographic scan of her chest, abdomen, and pelvis showed no evidence of tumors. She received methylprednisolone at a dose of 1 g per day for 5 days. Steroids were tapered down gradually as her symptoms relieved. Mycophenolate mofetil was added as the continued immunotherapy. The follow-up is still going on.

### Case 2

A girl in her 20s was admitted to our hospital with psychosis, behavioral change and complex partial seizure over a period of 5 months. A resection of nevus on her forehead was performed about 2 weeks before her admission, which was intradermal nevus on the histopathology. The brain MRI was unremarkable and the EEG demonstrated generalized slow activity. The CSF analysis revealed the WBC count was 66/μL with 99 lymphocytes and 1 % monocytes, and the protein concentration level was 72 mg/dL. The same tests with serum and CSF for available antibodies to cell-surface antigens were performed as previously described. High titres of anti-NMDAR antibodies were both detected in the CSF and serum. The screening tests for an ovarian teratoma and other tumors were all negative. She received one course of intravenous immunoglobulin, 2 g/kg, divided into 5 days, followed with intravenous methylprednisolone at an initial dosage of 80 mg per day. Gradually, her psychotic symptoms resolved and she was able to follow simple commands and talk to her parents. The test for NMDAR antibody in CSF became negative afterwards while that in serum remained positive.

## Discussion and conclusions

Anti-NMDAR encephalitis represents a characteristic immune-mediated and treatment responsive neuropsychiatric syndrome, with and without a tumor association. Dalmau et al. [1] reported the presence of an ovarian teratoma in over 50 % of anti-NMDAR encephalitis patients. The tumor that expresses NMDA receptors may trigger the antibody response against NMDA receptors by ectopically expressing neuronal antigens and contribute to breaking immune tolerance [2, 3]. However, they also indicated that 41 % of patients with anti-NMDAR encephalitis do not have a clinically detectable tumor. Therefore, the underlying causes in tumor-negative patients are still largely unknown. Our cases provided an observational link between anti-NMDAR encephalitis and resection of nevi, which may provide insights into the trigger factor of immune response in non–tumor associated anti-NMDAR encephalitis.

NMDA receptors are a class of ionotropic glutamate receptors (iGluRs) that are essential for neuronal development, synaptic plasticity and cell survival. NMDA receptors are obligate heterotetramers comprising of a combination of NR1, the obligatory subunit for a functional receptor, and structurally related NR2 subunits [4]. Studies have demonstrated the NR1 subunit of NMDA receptor is also expressed on human keratinocytes, using immunohistochemistry [5, 6]. Hoogduijn et al. found that cultured melanocytes express the NMDA receptors 2A and 2C [7]. Therefore, we postulate that the nevus cell may express NMDA receptor or other mimic cross-reactive antigens on the cell surface. Exposure of certain antigens caused by nevi excision may trigger an autoimmune response and generation of anti-NMDAR antibodies, resulting in encephalitis. This suggested a potential site of antigen exposure triggering the immune response in non-tumor associated anti-NMDAR encephalitis, which may lend support to elucidating the underlying immunopathological mechanisms. Further studies were expected for investigating the expression of NMDA receptor on nevus cell and evaluating the validity of this hypothesis.

## Consent

Written informed consent was obtained from the two patients for publication of this case report and any accompanying images. Copies of the written consents are available for review by the Editor-in-Chief of this journal.

## Abbreviations

MRI: Magnetic resonance imaging
EEG: Electroencephalogram
CSF: Cerebrospinal fluid
WBC: White blood cell
NMDAR: N-methyl-D-aspartate receptors
LGI1: Leucine-rich glioma inactivated 1
CASPR2: Contactin-associated protein 2
GABABR: γ-amino-butyric acid-B receptor
AMPAR: Alpha-amino-3-hydroxy-5-methyl-4-iso-xazolepropionic acid receptors
iGluRs: Ionotropic glutamate receptors
